# Collect Once, Use Many Times: Attaining Unified Metrics for Tuberculosis Preventive Treatment for People Living With HIV

**DOI:** 10.2196/27013

**Published:** 2021-04-30

**Authors:** Rena Fukunaga, David Lowrance, Adam MacNeil, Teeb Al-Samarrai, Joseph Cavanaugh, Annabel Baddeley, Catherine Nichols, Meaghan Peterson, Sevim Ahmedov, Vindi Singh, Celeste Gracia Edwards, Suman Jain, Anand Date, Susan A Maloney

**Affiliations:** 1 Centers for Disease Control and Prevention Atlanta, GA United States; 2 The Global Fund to Fight AIDS, Tuberculosis and Malaria Geneva Switzerland; 3 Office of the US Global AIDS Coordinator Washington, DC United States; 4 Walter Reed Army Institute of Research Silver Spring, MD United States; 5 World Health Organization Geneva Switzerland; 6 US Agency for International Development Washington, DC United States

**Keywords:** tuberculosis preventive treatment, monitoring and evaluation, people living with HIV, HIV, TB, infectious disease, preventative treatment

## Abstract

The World Health Organization (WHO) recommends providing tuberculosis preventive treatment (TPT) to all persons living with HIV and to all household contacts of persons with bacteriologically confirmed pulmonary tuberculosis disease. Regrettably, the absence of a harmonized data collection and management approach to TPT indicators has contributed to programmatic challenges at local, national, and global levels. However, in April 2020, the WHO launched the Consolidated HIV Strategic Information Guidelines, with an updated set of priority indicators. These guidelines recommend that Ministries of Health collect, report, and use data on TPT completion in addition to TPT initiation. Both indicators are reflected in the WHO’s list of 15 core indicators for program management and are also required by the US President’s Emergency Plan for AIDS Relief’s Monitoring, Evaluation, and Reporting (MER) guidance. Although not perfectly harmonized, both frameworks now share essential indicator characteristics. Aligned indicators are necessary for robust strategic and operational planning, resource allocation, and data communication. “Collect once, use many times” is a best practice for strategic information management. Building harmonized and sustainable health systems will enable countries to successfully maintain essential HIV, tuberculosis, and other health services while combatting new health threats.

## Commentary

Tuberculosis (TB) is the leading cause of death from a single infectious disease, with 1.2 million annual deaths worldwide and 10 million persons with incident TB estimated in 2019 [1]. To prevent TB disease in persons infected with HIV and at high risk for disease progression, the World Health Organization (WHO) recommends providing tuberculosis preventive treatment (TPT) to all persons living with HIV (PLHIV) and to all household contacts of persons with bacteriologically confirmed pulmonary TB disease [2].

At the 2018 United Nations High-Level Meeting on TB, member states committed to ambitious targets for TPT scale-up, supporting the long-term goal of ending the global TB epidemic by 2030. This pledge aims to provide TPT to at least 30 million people by 2022 (including 6 million PLHIV, 4 million children <5 years, and 20 million other household contacts of people affected by TB) [3,4]. Concomitantly, the US President’s Emergency Plan for AIDS Relief (PEPFAR) committed to providing a course of TPT to all 13.6 million PLHIV on antiretroviral therapy (ART) supported by PEPFAR by 2021 [5]. The WHO’s multisectoral TB accountability framework and HIV strategy also hold governments and stakeholders responsible for accelerating progress to end the TB epidemic and reduce TB-associated mortality among PLHIV including meeting the TPT targets [6]. In concert with these commitments, the Global Fund urges high TB/HIV burden countries to incorporate TPT into their funding requests and matches funds to incentivize country allocations for TPT scale-up among PLHIV [7].

Global reporting for 2019 showed gains in TPT scale-up among PLHIV, with 75 countries reporting initiation of TPT for 3.5 million individuals, up from 1.8 million in 2018 [1]. Examples of successful TPT expansion [8] demonstrate how resource-limited countries can overcome barriers, including modifying national and subnational health information systems (HIS) and monitoring and evaluation (M&E) processes to accommodate TPT data collection and reporting [9]. However, these modifications remain more of an exception than the rule, as the absence of a harmonized data collection and management approach to TPT indicators has contributed to programmatic challenges at local, national, and global levels. This includes challenges in identifying impactful global health trends (a consequence commonly seen with data silos), in ensuring timely communication between programmatic stakeholders, and in enhancing the usability of the data in programmatically meaningful ways. Data reported to the WHO and PEPFAR entail parallel and varying reporting periods, indicator definitions, and partner engagement; harmonizing these efforts could help to decrease reporting burden and unify data metrics for programmatic decision making.

In April 2020, the WHO launched the Consolidated HIV Strategic Information Guidelines*,* with an updated set of priority indicators [10]. These guidelines recommend that Ministries of Health collect, report, and use data on TPT completion in addition to TPT initiation. Both TPT indicators are reflected in the WHO’s list of 15 core, or highest priority, indicators for program management and monitoring and are also required by PEPFAR’s Monitoring, Evaluation, and Reporting (MER) guidance [11]. Although not perfectly harmonized, both frameworks now share essential indicator characteristics (Table 1). Global Fund’s modular performance framework uses the TPT initiation indicator to assess grant performance; while this alignment is only partial, it encourages countries to report TPT data as recommended by the WHO.

The ability to strengthen program management through improved data cannot be overstated. Robust data allow programs to identify geographic and sociodemographic differences in service coverage and quality and help ensure that no one is left behind. Aligned indicators are also necessary for robust strategic and operational planning, resource allocation, and data communication. Eliminating redundancies in the TPT data collection and management process will allow health care workers, data clerks, and TB and HIV program managers to reallocate their time toward optimizing service delivery and scale-up efforts, thereby becoming more efficient and effective. Simplified data collection methods may also reduce data entry errors and delays in reporting, although these benefits will not accrue immediately. National HIV and TB programs, which typically operate independently and use separate data systems, can strengthen their contributions by harmonizing metadata, M&E tools, and digital data systems, making HIS interoperable across health sector programs, training staff on new data collection requirements, and capitalizing on movements toward primary and universal health care.

“Collect once, use many times” is a best practice for strategic information at local, national, and global levels. Application of this principle includes standards-based indicator alignment and coordinated resource allocation for national M&E tools and HIS, along with concomitant coordination at the global level. It will eliminate parallel reporting systems and allow for the creation of a harmonized data set for use by partners at all levels. Such resilient, harmonized, and sustainable health systems will enable countries to successfully maintain essential HIV, TB, and other health services while combatting new health threats.

**Table 1:** A comparison of the World Health Organization and US President’s Emergency Plan for AIDS Relief’s global tuberculosis/HIV indicators^a^.

Indicators	World Health Organization (and Global Fund)	US President’s Emergency Plan for AIDS Relief (PEPFAR)
Document name (version)	Consolidated HIV Strategic Information Guidelines: Driving Impact Through Programme Monitoring and Management (April 2020)	Monitoring, Evaluation, and Reporting Indicator Reference Guide – MER 2.5 (September 2020)
Description	Proportion of patients receiving ART who started on a standard course of TPT in the previous reporting period who completed therapy	Proportion of patients receiving ART who started on a standard course of TPT in the previous reporting period who completed therapy
Numerator (TPT completion)	Number of PLHIV on ART who completed a course of TPT among those who initiated TPT	Among those who started a course of TPT in the previous reporting period, the number that completed a full course of therapy. For continuous IPT programs, this includes the patients who have completed the first 6 months of IPT, or any other standard course of TPT, such as 3 months of weekly isoniazid and rifapentine, or 3-HP.
Denominator (TPT initiation)	Number of eligible PLHIV on ART who initiated TPT	Number of patients on ART who were initiated on any course of TPT during the previous reporting period
Data elements (disaggregates)	Descriptions: Sex: male, female, transgender Age bands: <15 years, ≥15 years Type of TPT regimen ART initiation: <12 months on ART, ≥12 months on ART	Age/sex by ART start descriptions: Newly enrolled on ART: these individuals initiated TPT within 6 months of being enrolled on ART Previously enrolled on ART: these individuals initiated TPT at least 6 months (or longer) after being enrolled on ART Age/sex bands: <15 years female/male, ≥15 years female/male, unknown age female/male
Reporting level	Facility	Facility
Reporting frequency	Quarterly, semiannually, and/or annually	Semiannually, with results encompassing achievements from October 1-March 31 and April 1-September 30
Most recent changes	New indicator in 2020.	No changes between MER v2.4 to v2.5.

^a^ART: antiretroviral therapy; IPT: isoniazid preventive therapy; MER: monitoring, evaluation, and reporting guidance; PLHIV: persons living with HIV; TPT: tuberculosis preventive treatment.

## Disclaimer

The authors alone are responsible for the views expressed in this article and they do not necessarily represent the views, decisions or policies of the institutions with which they are affiliated.

## Abbreviations

ART: antiretroviral therapy
HIS: health information system
MER: Monitoring, Evaluation, and Reporting
M&E: monitoring and evaluation
PEPFAR: US President’s Emergency Plan for AIDS Relief
PLHIV: persons living with HIV
TB: tuberculosis
TPT: tuberculosis preventive treatment
WHO: World Health Organization
